# Endoscopic Resection of Esophageal Lymphangioma Incidentally Discovered

**DOI:** 10.1155/DTE.4.141

**Published:** 1998

**Authors:** M. Scarpis, M. De Monti, M. G. Pezzotta, D. Sonnino, D. Mosca, M. Milani

**Affiliations:** 1 Menaggio Community Hospital (Como, Italy) Department of General Surgery Digestive Endoscopy Service Italy; 2 Italian Emergency System S.S.U.Em 118 Menaggio Italy; 3 Lecco General Hospital Cytodiagnostic and Pathoanatomy Service Italy; 4 Via Derna 5 Milano 20132 Italy

## Abstract

A pedunculated lymphangioma of the esophagus was unexpectedly discovered during an endoscopic investigation performed for epigastric pain in a patient affected by diabetic arteriopathy treated with antiplatelet drugs. The patient neither complained of dysphagia nor other symptoms related to the presence of the lymphangioma which therefore can be considered as an endoscopic “incidentaloma”.

The lesion was removed endoscopically and a follow up, 6 months later, showed no scar or recurrence.

The authors present this case both for the extreme rarity of this lesion and for the evidence of low-medium grade dysplasia in the overlying mucosa, particularly since it is only case ever noted in literature.

This aspect suggests that, even if malignant degeneration of these lesions has never been observed, their endoscopic removal is recommended. However, when endoscopic procedures are not feasible, thoracotomic surgical exeresis should be only considered for obstructing and symptomatic lesions; an accurate endoscopic and bioptic follow up can be useful for asymptomatic lesions.


*Diagnostic and Therapeutic Endoscopy*, Vol. 4, pp. 141–147
Reprints available directly from the publisher
Photocopying permitted by license only

© 1998 OPA (Overseas Publishers Association)
Amsterdam B.V. Published under license
under the Harwood Academic Publishers imprint,
part of The Gordon and Breach Publishing Group.
Printed in Singapore.

### Endoscopic Resection of Esophageal Lymphangioma Incidentally Discovered: A Case Report

M. SCARPIS [a], M. DE MONTI [b,*], M.G. PEZZOTTA [c], D. SONNINO [a], D. MOSCA [a] and M. MILANI [c]

[a] *Menaggio Community Hospital (Como, Italy), Department of General Surgery, Digestive Endoscopy Service;* [b] *Italian Emergency System, S.S.U.Em 118 Menaggio;* [c] *Lecco General Hospital, Cytodiagnostic and Pathoanatomy Service, Italy*

*(Received 6 May 1997; Revised 14 July 1997; In final form 25 August 1997)*

**A pedunculated lymphangioma of the esophagus was unexpectedly discovered during an endoscopic investigation performed for epigastric pain in a patient affected by diabetic arteriopathy treated with antiplatelet drugs. The patient neither complained of dysphagia nor other symptoms related to the presence of the lymphangioma which therefore can be considered as an endoscopic "incidentaloma".**

**The lesion was removed endoscopically and a follow up, 6 months later, showed no scar or recurrence.**

**The authors present this case both for the extreme rarity of this lesion and for the evidence of low-medium grade dysplasia in the overlying mucosa, particularly since it is only case ever noted in literature.**

**This aspect suggests that, even if malignant degeneration of these lesions has never been observed, their endoscopic removal is recommended. However, when endoscopic procedures are not feasible, thoracotomic surgical exeresis should be only considered for obstructing and symptomatic lesions; an accurate endoscopic and bioptic follow up can be useful for asymptomatic lesions.**

*Keywords:* Esophageal neoplasm, Lymphangioma, Endoscopic incidentaloma

### INTRODUCTION

Lymphangiomas are a rare form of benign vascular tumors. Following Wegener's classification [1,2], they can be subdivided into three groups: simple, cavernous, or cystic. These lesions can be single or multiple in the so called "lymphangiomatosis" [3].

Lymphangioma can occur in several anatomical regions: neck (75%), axilla (20%), mediastinum, bone and retroperitoneum (5%) [3]. Lymphangiomas of the liver and of the spleen have been rarely noted (they usually have a worse and invasive course). Moreover, malignant degeneration has never been observed [4].

* Corresponding author. Via Derna 5, 20132 Milano, Italy. Tel.: +39 2 26 11 13 44; +39 330 23 05 55. Fax: +39 344 30596.

Lymphangiomas of the gastrointestinal tract are even more rare: Gangl [5] collected only 32 gastrointestinal lymphangiomas in the literature: the most commonly affected area is the colon, followed by duodenum and stomach. To date, only eight cases of esophageal lymphangiomas have been observed [6]. In fact, leiomyomas are the most common benign tumor of this organ (59%) [7,8]. Instead vascular tumors represent only 2% of all esophageal benign neoplasms, but most of them are hemangiomas [9,10].

The Authors present a case of esophageal lymphangioma incidentally discovered, both because of the extreme rarity of this lesion and to report its successful endoscopic removal and, to provide evidence of a low-medium grade dysplasia in the epithelium never before noted.

### CASE REPORT

*S.B.F., white man,* 64 *year old, weight* – 81 kg, *height* – 171 cm. He was admitted to our department with the diagnosis of peripheral occlusive arterial disease at Fontaine's IV grade. He smoked approximately 30 cigarettes, and consumed roughly $\frac{1}{2}$–1 litre of wine a day. The patient's father had suffered stomach cancer.

Upon admittance, the patient complained of persistent epigastralgia. Consequently, he was submitted for an esophagogastroscopy which showed the presence of two pyloric ulcers measuring roughly 5 mm in diameter and a positive test for Helicobacter Pylori. Additionally, a pedunculated lesion of 15 mm in diameter was discovered in the lower third of his esophagus (Fig. 1): thus, an endoscopic polypectomy was performed with a diathermic loop. When the patient was released from the hospital, he was given a two-week daily prescription of Omeprazole 40 mg and Amoxicillin 2 g for the elimination of Helicobacter Pylori.

Histologic examination exhibited the presence of a lymphangioma with focal low-medium grade dysplasia in the epithelium (Figs. 2, 3).

After 6 months, another esophagogastroscopy was performed, which showed complete healing

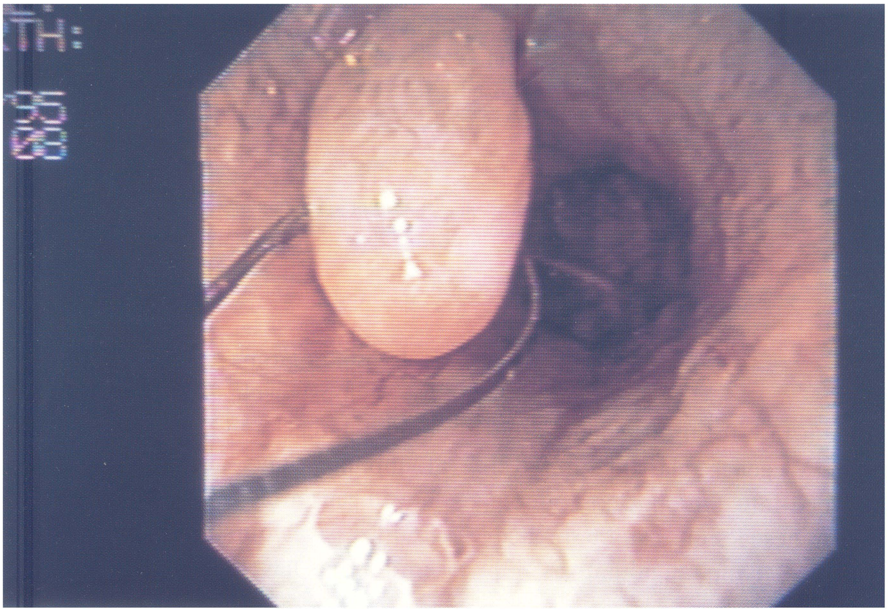

FIGURE 1 Esophageal lymphangioma: endoscopic appearance. The diathermic loop for the operative procedure is evident.

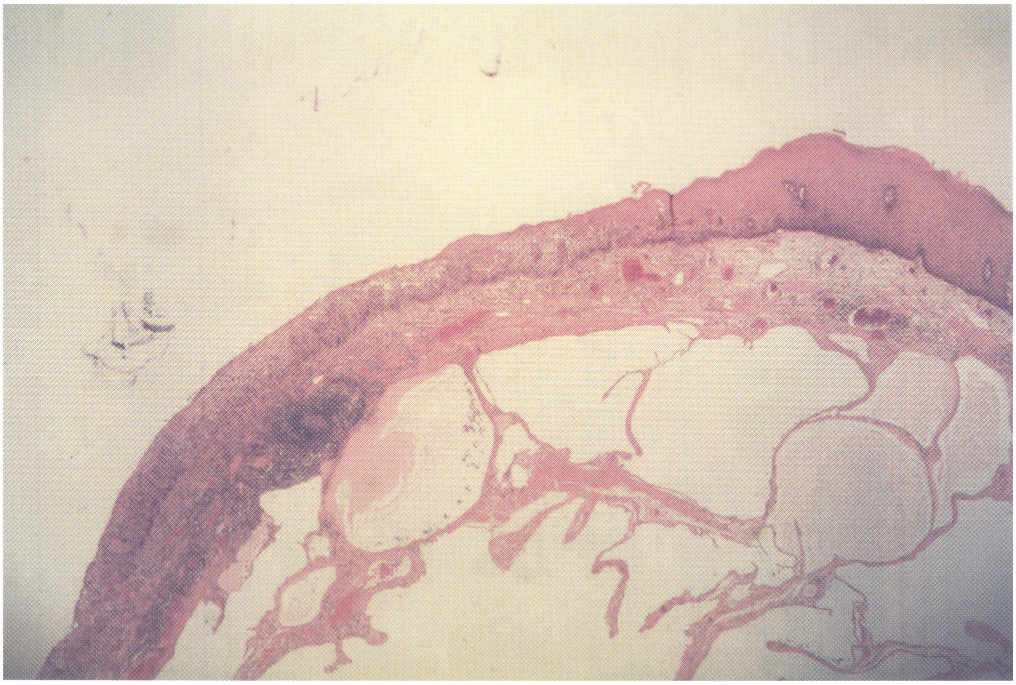

FIGURE 2 Esophageal lymphangioma. Normal (right) and dysplastic (left) esophageal epithelium, enlarged lymphatic vessels and lymphoid follicle are appreciated at this magnification (EE, 25X).

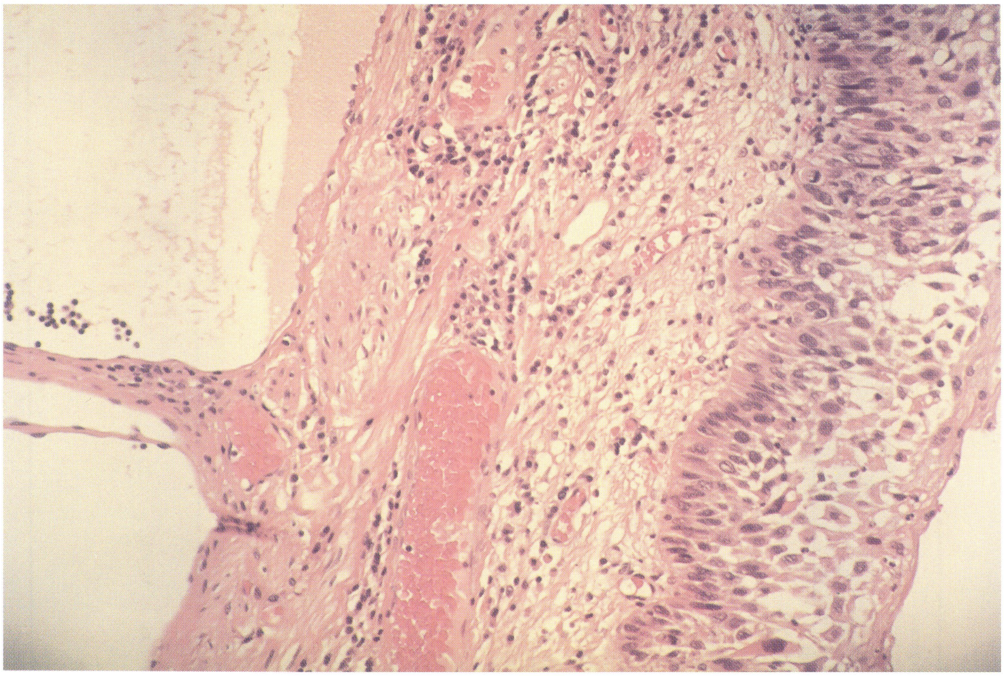

FIGURE 3 Esophageal limphangioma. Enlarged lymphatic vessels without blood, mild chronic inflammation and spongiotic dysplastic epithelium are demonstrated (EE, 160X).

TABLE I

| Age (y) | Sex | Symptoms | Diagnosis | Location | Size (mm) | Sessile/ polypoid | Stenosis | Capillary dilatation | Redness of mucosa | Erosion of mucosa | Endoscopic biopsy | Therapy | Histological examination | Dysplasia | Concomitant lesions | Authors | Year | Reference | Follow up |
|---|---|---|---|---|---|---|---|---|---|---|---|---|---|---|---|---|---|---|---|
| — | — | — | Postmortem examination | — | — | — | — | — | — | — | — | — | — | — | — | Schmidt *et al.* | 1961 | 11 | — |
| 62 | F | Peptic disease related | EGDS | Middle (30 cm from dental arch) | 50 | Sessile | YES | NO | NO | NO | Deep biopsy with Edder–Hufford esophagoscope: lymphangioma | Conservative therapy | — | NO | Gastric ulcer | Brady and Milligan | 1973 | 12 | 18 months: no dysphagia or odynophagia |
| 64 | M | Peptic disease related | EGDS | Middle (30 cm from dental arch) | 10 | Polypoid | NO | NO | NO | NO | NO | Endoscopic | Submucosal lymphangioma | NO | Gastric ulcer | Armengol-Miro *et al.* | 1979 | 14 | — |
| 46 | M | Increasing dysphagia and occasional vomiting after meals | Upper gastrointestinal Rx series | Lower | Not referred | Sessile | YES | NO | NO | NO | NO | Surgical (Right thoracotomy) | Cavernous lymphangioma | NO | Achalasia (Mecholyl test positive) | Tamada *et al.* | 1980 | 20 | — |
| 55 | M | Disfagia (suspicion of achalasia) | Upper gastrointestinal Rx series | Middle | 20 | Sessile | YES | NO | NO | NO | NO | Surgical | Cavernous lymphangioma | NO | — | Kralik *et al.* | 1982 | 21–22 | — |
| 58 | M | Epigastric burning and reflux symptoms in the substernal area. Later intermittent dysphagia | EGDS | Lower | 15 | Sessile | NO | NO | NO | NO | Oesophagitis | Endoscopic | Submucosal lymphangioma | NO | Hiatus hernia | Liebert | 1983 | 13 | — |
| 66 | F | Chest pain, occasional heartburn for 2 months | EGDS | Middle | 20 | Sessile | YES | NO | NO | NO | NO | Surgical | Lymphangioma | NO | Chronic coronary artery disease | Castellanos *et al.* | 1990 | 6 | 2 months: persistence of occasional heartburn |

TABLE I *(Continued)*

| | | | | | | | | | | | | | | | | | | |
|---|---|---|---|---|---|---|---|---|---|---|---|---|---|---|---|---|---|---|
| 19 months | F | Wheezing, dyspnea, respiratory distress, vomiting unrelated to mealtime | Frontal chest Rx | High | 45 mm × 12 cm of length | Polypoid | YES | YES | YES | YES | NO | Emergency surgery for respiratory distress and sepsis | Mixed hemangioma and cystic lymphangioma | NO | NO | Farley and Klionsky | 1992 | 15 | Not specified the time, but it is referred the child remains in good health and is gaining weight |
| 55 | M | Occasional, slight, retrosternal burning unrelated to swallowing | Upper gastorintestinal Rx series | Middle (25 cm from dental arch) | 40 | Sessile | NO | NO | NO | NO | Chronic oesophagitis | Surgical. Combined right thoracotomy (5° i.s.) and i.o. endoscopy | Cavernous lymphangioma | NO | Chronic oesophagitis | Yoshida *et al.* | 1994 | 4 | Two week later: normal morphology and function of oesophagus |
| 65 | M | Peptic disease related | EGDS | Lower | 13 | Polypoid | NO | NO | NO | NO | NO | Endoscopic | Lymphangioma with focalised low-medium grade dysplasia of the mucosa | Mucosa | 2 juxtapiloric gastric ulcers; Helicobacter test positive | Scarpis *et al.* | 1995 | Present case | 6 months: normal morphology and function of oesophagus; persistence of gastric ulcers |

All cases published in the official scientific literature.

of the esophageal mucosa. In addition, the area where the polypectomy had been previously performed was no longer visible, and the two gastric ulcers were still present, based on a positive test for Helicobacter Pylori. Consequently, the patient was given a two-week prescription of amoxicillin, omeprazole and metronidazole (250 mg to be taken four times daily). The subsequent clinical examination, four months later, showed that the epigastralgia had disappeared. Based on these results, no further endoscopic exams were carried out.

### DISCUSSION

Schmidt [11] described the first esophageal lymphangioma, discovered during an autopsy, in 1961. Then Brady [12] described the first endoscopic finding of an esophageal lymphangioma. To-date, only 8 cases have been published in the official scientific literature (Table I). Of these, only two were removed endoscopically [13,14]. Another case occurred in a 19 month old baby: it was surgically removed and the histological examination showed a mixed hemangioma and cystic lymphangioma [15].

Symptoms vary depending on the location, dimension, and degree of obstruction. In fact, the lesion can be completely asymptomatic or can cause dysphagia and odynophagia; in cases where patients have complained of chest pain in the mid sternal area, lymphangioma was also present with either esophagitis, hiatal hernia, gastric ulcers or coronaropathy: for this reason, the correlation to the lymphangioma is difficult to determine.

Lymphangiomas can be considered hamartomas originating from deep lymphatic structures and their pathogenesis can be related to the cystic dilatation of the enclosed lymphatic tissue, which preserves its potential endothelial growth. On gross examination, the lesions are pale, smooth, with multicystic cut surface and exude clear yellowish fluid. Histologically, the masses are composed of a loose myxoid stroma and variably sized enlarged channels lined by lymphatic endothelial cells. There is no blood within the vascular space.

The hypothesis that lymphangiomas could be functioning lymphatic vessels is based on the fact that lymphographies of retroperitoneal lesions demonstrated an opacification of the cavities within 48 h [16].

Lymphangioma can occur in every age, but several authors have reported a higher incidence during childhood [17–19].

Esophageal lymphangioma appears as a pale, translucent and cystic lesion which softly deforms under the pressure of the endoscope. Its endoscopic aspect can sometimes be difficult to differentiate from submucosal sessile lesions as esophageal varices [4]: for this reason, several authors consider endoscopic biopsy as dangerous [20]. Radiotherapy appears to be inadequate for these neoplasms [6], and endoscopic resection, when possible, in our opinion is recommended. In fact, even if malignant degeneration has never been observed, our case gives evidence, for the first time in literature, of focal low-medium grade dysplasia in the mucosa overlying the pedunculated lesion. Thoracotomic surgical resection of lesions, which are not endoscopically resectable, should be indicated only after an accurate evaluation of risks and benefits, with consideration of possible conservative therapy of asymptomatic and non-obstructing lesions with an endoscopic and bioptic follow-up.

### *References*

[1] Wegener, G. Lymphangiome. *Arch. Klin. Chir.* 1877; **20**: 641.

[2] Pyatt, R.S., Williams, E.D. and Clark, M. CT diagnosis of splenic cystic lymphangiomatosis. *JCAT* 1981; **5**: 446–8.

[3] Richetta, L., Cirillo, S., Isolato, G., Cesarani, F. and Avataneo, T. Aspetto della linfangiomatosi cistica alla Risonanza Magnetica. A proposito di un caso. *Radiol. Med.* 1993; **85**: 135–38.

[4] Yoshida, Y. *et al.* Lymphangioma of the oesophagus: a case report and a review of the literature. *Thorax* 1994; **49**: 1267–68.

[5] Gangl, A., Polterauer, P., Krepler, R. and Kumpan, W. A further case of submucosal lymphangioma of the duodenum diagnosed during endoscopy. *Endoscopy* 1980; **12**: 188–90.

[6] Castellanos, D. *et al.* Esophageal lymphangioma: case report and review of the literature. *Surgery* 1990; **108**: 593–4.
[7] Persichetti, P. *et al.* Esophageal leiomyoma: review of the literature and a case record from our institute. *Minerva Chir.* 1984; **11**: 855–62.
[8] Gutierrez, M.T. *et al.* Tumores benignos de esofago: leiomioma esofagico. *Rev. Esp. Oncol.* 1985; **32**: 307–14.
[9] Watson, R.R., O'Connor, T.M. and Wilson, W. Solid benign tumors of the esophagus. *Ann. Thorac. Surg.* 1967; **4**: 80–91.
[10] Plachta, A. Benign tumors of the esophagus: review of literature and a report of ninety-nine cases. *Am. J. Gastroenterol.* 1962; **38**: 639–52.
[11] Schmidt, H.W., Clagett, O.T., Harrison, E.G. Jr. Benign tumors and cysts of the esophagus. *J. Thorac. Cardiovasc. Surg.* 1961; **41**: 717–32.
[12] Brady, P.G. and Milligan, F.D. Lymphangioma of the esophagus. Diagnosis by endoscopic biopsy. *Dig. Dis.* 1973; **18**: 423–25.
[13] Liebert, C.W. Jr. Symptomatic lymphangioma of the esophagus with endoscopic resection. *Gastrointest. Endosc.* 1983; **29**: 225–6.
[14] Armengol-Miro, Jr. *et al.* Lymphangioma of the oesophagus. Diagnosis and treatment by endoscopic polypectomy. *Endoscopy* 1979; **3**: 185–9.
[15] Farley, T.J. and Klionsky, N. Case report. Mixed hemangioma and cystic lymphangioma of the esophagus in a child. *J. Pediatr. Gastroenterol. Nutr.* 1992; **15**: 178–80.
[16] Guerin, E., Babin, C. and Moulle, P. Lymphangiome kystique rétropéritoneal: diagnostic préopératoire. A propos d'un cas. *J. Radiol.* 1987; **68**: 693–5.
[17] Harkins, G.A. and Sabiston, D.C. Lymphangioma in infancy and childhood. *Pediatr. Surg.* 1960; **47**: 811–22.
[18] Leonidas, J.C., Brill, P.W. and Bahn, I. Cystic retroperitoneal lymphangioma in infants and children. *Radiology* 1978; **127**: 203–8.
[19] Van Cauwelaert, P.H. and Gruwez, J.A. Experience with lymphangioma. *Lymphology* 1978; **11**: 43–48.
[20] Tamada, R., Sugimachi, K., Yaita, A., Inokuchi, K. and Watanabe, H. Lymphangioma of the esophagus presenting symptoms of achalasia. A case report. *Jap. J. Surg.* 1980; **10**: 59–62.
[21] Kralik, J. and Curik, R. Lymfangiom jicnu. *Cesk Otolaryngol.* 1982; **31**: 306–9.
[22] Kralik, J. Lymphangiome der Speiserohre und des Magens. *Zentralbl. Chir.* 1983; **108**: 272–5.

